# Promoting healthy practices among schools and children in rural bangladesh: a randomised controlled trial of skill-based health education

**DOI:** 10.1186/s12889-024-20787-0

**Published:** 2024-11-27

**Authors:** Makiko Omura, Mohini Venkatesh, Ikhtiar Khandaker, Md. Ataur Rahman

**Affiliations:** 1 Department of Economics, Faculty of Economics, Meiji Gakuin University, Tokyo, Japan; 2 Department of Education and Child Development, International Programs, Save the Children Federation, Inc., Fairfield, Connecticut USA; 3 Care Bangladesh, Dhaka, Bangladesh; 4 Maternal & Child Wasting Project, Result for Development (USA), Dhaka, Bangladesh

**Keywords:** Cluster randomised-controlled trial (RCT), Field experiment, Cross-cutting/factorial design, Skill-based health education (SBHE), Primary school hygiene, Child health practices, Behaviour change, Spillover effects, Cost-effectiveness, Bangladesh

## Abstract

**Background:**

Poor child health and hygiene practices are persistent issues in resource-constrained settings, particularly in low-income countries. This study assessed the impact of skill-based health education (SBHE) on school and child hygiene practices in rural Bangladesh.

**Methods:**

A cluster-randomised-controlled intervention with cross-cutting/factorial design was conducted in 180 randomly selected primary schools, stratified by school type, in Jhenaidah District, Bangladesh. Weekly SBHE sessions were delivered to half of the schools by locally recruited para-teachers for one year. A cross-cutting soap provision treatment was given monthly to half of the SBHE-treatment schools and half of the SBHE-control schools. Treatment assignment was masked to all baseline and endline surveyors. Data were collected at both the school and child levels, with child-level data aggregated at the school level. Outcome measures were grouped into five thematic families. The primary outcome families were *school hygiene practice & maintenance*, *school-aggregated child handwashing* and *school-aggregated child dentalcare*. Utilising the difference-in-differences estimator with seemingly unrelated regression, we estimated the average treatment effect for each family of multiple outcomes. The intervention spillover effect to neighbouring schools along with the time-period effect were also evaluated. The project’s cost-effectiveness was additionally assessed.

**Results:**

Our findings revealed that SBHE had a positive impact on primary outcomes related to healthy practices and behavioural changes, resulting in a 0.32SD improvement in school hygiene practices and maintenance (*p* < 0.001), a 0.47SD increase in child handwashing (*p* < 0.001), and a 0.43SD enhancement in child dentalcare (*p* < 0.01). Despite its imperfect implementation, the provision of soap itself showed no significant effect. Furthermore, significant spillover effects of healthy practices were observed in neighbouring non-treatment schools. The cost-effectiveness analysis indicated that our SBHE program was cost-effective.

**Conclusions:**

Our study provides compelling evidence of the positive impact of SBHE on school hygiene and child health practices in rural Bangladesh, with notable spillover effects. The cost-effectiveness analysis underscores the value of SBHE, affirming its potential as an effective intervention method in improving school health and hygiene practices in primary schools and beyond.

**Supplementary Information:**

The online version contains supplementary material available at 10.1186/s12889-024-20787-0.

## Background

The imperative to improve health outcomes in resource-limited settings particularly in low-income countries is well-recognised. Existing literature emphasises the potential of health education to foster healthier environment and habits, notably in school context [1, 2]. However, few rigorous evaluations of health education have been conducted, and these educational interventions were often found to be ineffective, especially when compared to direct provision of drugs and/or supplements, which tended to be cost-effective and provide immediate benefits [3–5].

Despite these findings, specific skill-based health education (SBHE) focusing on practical skills like handwashing and sanitisation [6–8], and oral hygiene education [9–11] has demonstrated significant positive outcomes. Additionally, simple but relevant information provision has proven effective in preventing HIV/AIDS infection compared to more general health education approaches [12, 13]. These findings suggest that efficacy of health education may be enhanced by focusing on context-relevant information and practical skill development.

Schools present a favourable environment for implementing SBHE due to the ability to reach many pupils simultaneously, leveraging both economies of scale and peer effects. Moreover, children are considered more amenable to behavioural change, making schools an ideal setting for such interventions. Nonetheless, challenges persist, primarily related to training and motivating teachers to deliver effective and contextually relevant health education [14–17]. Our study tackled these difficulties by employing para-teachers and mobile projectors to deliver SBHE, circumventing more traditional barriers such as teacher motivation, teacher skills and resource limitations. While our SBHE intervention did not provide any hygiene infrastructure, it was designed to promote better maintenance practices of existing facilities. This approach addressed the observed inadequacies in managing hygiene infrastructure in low-resource contexts, where facilities often fell into disrepair and underutilisation, resulting in shortened lifetime after their provision [7]. Latrines in Bangladeshi primary schools were generally lacking in numbers, unclean and/or locked up. Even the already poorly available infrastructural resources were poorly managed and not fully utilised—newly built latrines became unusable and discarded after several years. Soaps were unavailable or were available only in teachers’ room upon request. Yet, a cluster-randomised controlled trial (RCT) study aiming to improve shared toilets maintenance in urban slums in Dhaka, Bangladesh, demonstrated that behaviour change, coupled with the provision of simple, low-cost goods led to better maintenance of the hygiene infrastructure [18]. The finding suggests that altering default practices towards better maintenance can achieve a positive impact at a low cost and in a short time, with the potential for sustainable effects.

This study hypothesises that SBHE, enhanced with innovative methods such as the use of trained para-teachers and mobile projectors, will be more effective than traditional methods in improving health-related school environment, pupil’s healthy practices, and their overall health in resource-limited settings. Further, we aim to assess the cost-effectiveness of this approach and its spillover effects on non-intervention schools.

## Methods

### Study design

The project applied a treatment-control pre-post evaluation based on a cross-cutting/factorial randomisation design of two interventions, SBHE (HE) and a soap-provision (SP), for one year. The unit of intervention was school. We selected 180 schools from a total of 204, stratifying them by type—government primary schools (GPS) and registered non-government primary schools (RNGPS). These schools were then randomised into treatment and control groups for each intervention. Each intervention encompassed 90 schools, with half of each intervention group receiving both HE and SP in a cross-cutting manner. Hence, a quarter of the schools received only the HE-treatment, another quarter received both HE- and SP-treatments, a further quarter received just the SP-treatment, and the final quarter served as the pure control group, receiving no treatments. The SP-intervention supplied soaps directly to schools and selected students, without accompanying health education, to isolate the effect of goods provision on healthy behaviours like handwashing. The SP-treatment had another role in mitigating possible Hawthorne effects among the HE-treatment schools. Hawthorne and John Henry effects refer to changes in behaviour of treatment and control groups, respectively, due to the sheer fact of evaluation taking place. In order to mitigate John Henry effects among the pure controls, we provided game boards to these schools as non-intrusive compensatory goods at the beginning of the project, which would not have affected the measurably of our intervention.

### Randomisation

A cross-cutting/factorial HE-SP treatments were randomly assigned to 180 schools stratified by two school-types. Thus, four groups (HE, SP, HESP, and control) with 45 school each were randomly chosen using an Excel random classification formula. The random assignment of schools to treatment and comparison groups was intended to ensure that the schools in each group were comparable in all significant respects except for the treatment status. Thus, the randomisation process was repeated until no statistical differences were observed in baseline school characteristics (student size; attendance rate) and school-level outcomes (hygiene infrastructure availability and status; hygiene practice) between the groups. The randomisation was done after the beginning of academic year in Bangladesh; thus, treatment status should not have affected the choice of school by families. Random selection of children within schools was based on seat placement, using the pre-determined randomly selected seat numbers. Surveyors were masked about the treatment status in both baseline and endline surveys.

### Setting and participants

The project took place in two sub-districts (*upazila*) of Jhenaidah District (*zila*), which was a target district of Save the Children (SC)’s PROTEEVA (Promoting Talent Through Early Education) project. Our intervention was implemented in two non-PROTEEVA sub-districts, Moheshpur and Kodchandpur with the area of 21.16km^2^ and 20.16km^2^, respectively. These neighbouring sub-districts were identified as needy areas, and they provided logistical advantages due to existing SC offices and local NGO partnerships. To avoid study contamination, there was no overlap between our project and PROTEEVA-targeted schools/communities. Data collection occurred over four months for baseline (13 October 2011 to 29 November 2011) and five months post-intervention for endline (7 April 2013–6 July 2013), with additional follow-up data verification. Among 204 primary schools in the area (103 GPS, 101 RNGPS), 90 GPS and 90 RNGPS were chosen for the project.

With 180 schools, the sample size calculation at the time of the baseline survey was based on the expected improvement of 0.15 standardised effect size in child-level indicators, which was conservative, detected with 80% power and 5% significance level, assuming the school-level covariates to explain 2.5% of the variance. The sample size calculation utilised the school intra-cluster correlation of 0.058, which was derived from SC’s baseline survey data in a different area in Bangladesh in which SC implemented a school health and nutrition project. A sample size of 34 students per schools would have been sufficient. However, to account for partial compliance and/or attrition, estimated at 20%, we included 10 students, five of each sex, in each grade 1–4, totalling 40 students per school at baseline.

**Fig. 1:**
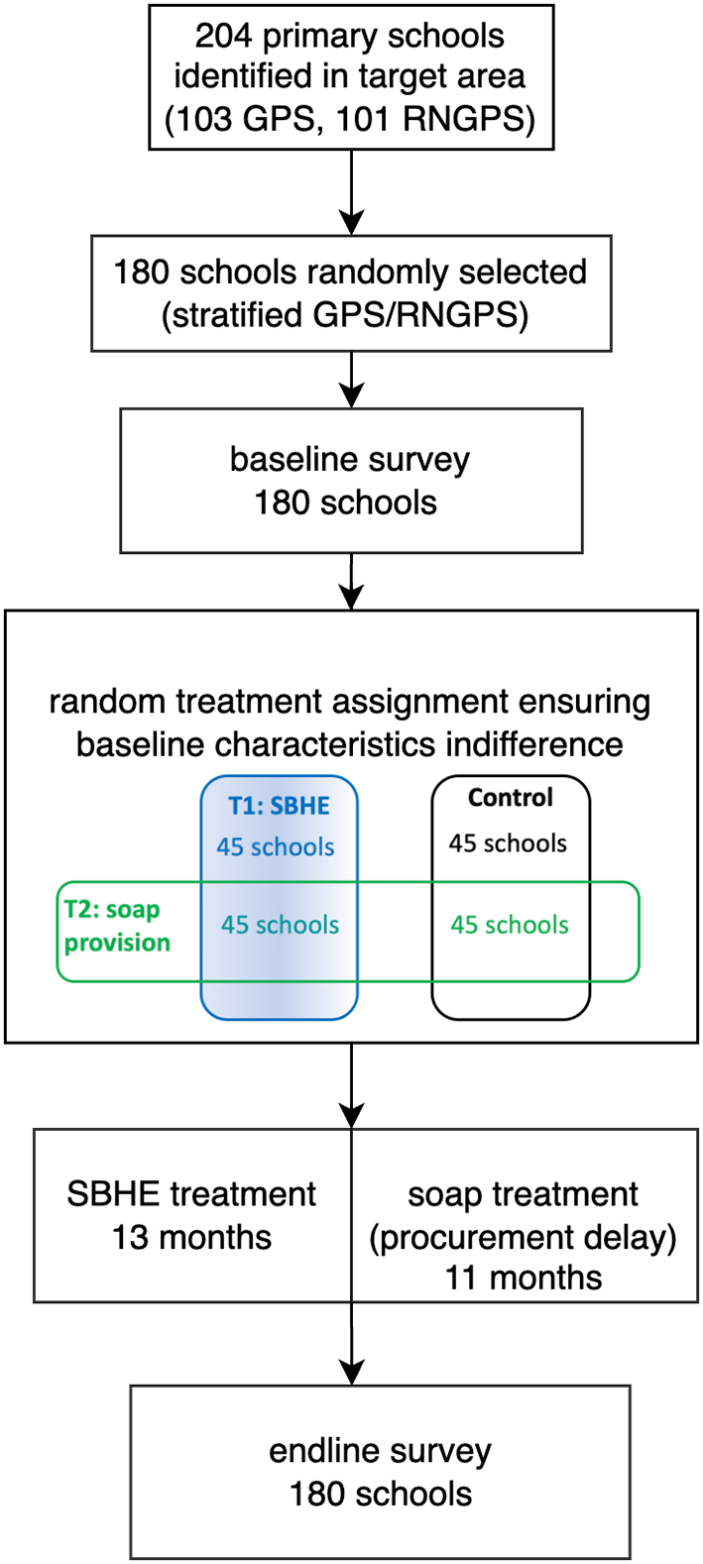
Project flow with randomisation design

Primary schools in Bangladesh are composed of grades 1–5. Because the sample targets were those who were in primary school during the intervention period, the baseline survey was conducted for grades 1–4, totalling 7,200 pupils. These children would be in grades 2–5 at the time of intervention and grades 3–6 at the time of the endline survey unless they repeated a grade or dropped out. Additionally, for the endline survey, we included grade 1or 2 pupils who were in grade 1 at the time of the intervention, totalling 9,000 pupils. Consequently, at endline, data were collected from grades 1–6, where grade 1 or 2 signified those who had been pre-primary at baseline, and grade 6 students had already graduated from primary school by then. Children surveyed at baseline were also surveyed at endline, with replacement participants included for those who had attritted. The project profile depicting the participant flow with randomisation design is provided in Fig. 1.

For school data, interviews were conducted to headteachers, and observational data were collected with photographs. For child data, interviews and observational data were collected. All interviews and data collection used structured questionnaires [19]. Data were collected from the interviewees upon informed consent. This study was approved by the Research Integrity Review Board of the author’s institute and by the Ministry of Education, Directorate of Primary and Mass Education in Dhaka and in the District of Jhenaidah. Our field experiment was retrospectively registered with the American Economic Association’s registry for randomised controlled trials (AEARCTR-0004265) on 2 June 2019 due to its establishment post-experiment, and with the ISRCTN registry for clinical trial (No.18002856) on 17 November 2023, following the understanding of an expanded definition of clinical trials. This reflects our adherence to evolving research standards.

### Skill-based health education strategies

The SBHE treatment was designed with the following key features: (1) weekly health education sessions using participatory learning methods to promote habit-formation; (2) use of para-teachers instead of primary school teachers as the SBHE session facilitators, given their cost-effectiveness and high motivation vis-à-vis incentive problems expected among the incumbent teachers; and (3) use of mobile mini-projectors to enhance pedagogy and skills learning, and to motivate both students and teachers. The mini-projectors could be easily charged in advance which was recommended due to Bangladesh’s unreliable power supply.

The health education session consisted of 26 modules in seven themes; (1) personal hygiene, (2) sanitation, (3) safe water, (4) common illnesses, (5) nutrition, (6) first aid, (7) injury prevention (see Table 1 for details). The educational contents utilised images and video footages relevant to the Bangladeshi context and made appropriate for primary school pupils, such as *Meena* by UNICEF [20]. Learning-by-doing sessions covered techniques for handwashing, brushing teeth, making oral rehydration solution (ORS), using latrines, and cleaning latrines, among others. Footage taken by the project staff, such as a well-dressed male staff member demonstrating proper latrine cleaning, was used not only to instruct on the methods but also to help reduce prejudice against ‘dirty jobs.’ In addition to SBHE sessions, our project introduced a routine classroom/latrine cleaning scheme by pupils modelled after practices in Japanese public schools. Weekly SBHE was provided for one class period a week for each grade, using a class slot allotted for physical education (PE). PE classes were usually free time for children to play around. Para-teachers taught five classes a day at the same school each day, rotating among five different schools over the course of the week; thus, each para-teacher performed a total of 25 h of SBHE sessions per week.

**Table 1:** Twenty-six skill-based health education modules

Themes (no. of modules)	Modules
1. personal hygiene (3)	handwashing, hair and nail trimming, bathing, cloth-cleanliness, eye care; teeth-brushing; tooth decay
2. sanitation (3)	keeping premises clean and waste management; latrine cleaning; sanitary latrine use
3. safe water (3)	safe and unsafe water; water purification and preservation; arsenic problem and prevention
4. common illnesses (6)	fever; cold and cough; diarrhoea cause; diarrhoea prevention; causes and demerits of worms; deworming
5. nutrition (5)	balanced diet; symptoms of malnutrition; vitamin A; iodine; iron
6. first aid (3)	burns; cuts; fractures
7. injury prevention (3)	burns; drowning; road accidents

The project locally recruited and trained the para-teachers who were either recent college graduates or former NGO workers seeking employment. There were several reasons for recruiting them: (1) regular teachers were already over-burdened by their own curricula, and similar SC projects had encountered moral hazards when using incumbent teachers, even with additional payments; (2) younger job seekers were more flexible and eager to learn and teach the SBHE content, particularly because acquiring skill-based methods and learning to use mobile mini-projectors would enhance their human capital; (3) it was considered sustainable cost-wise even after the project, as the cost per para-teacher was 5,000 BDT per month, approximately 67 USD (at 1 USD = 74.66 BDT). This amount was slightly less than the regular GPS assistant teacher’s minimum salary of 5,900 BDT, translating to an affordable 1,000BT or 13.4 USD per school. Para-teachers underwent over 40 h of intensive participatory-training in SBHE by BRAC health education specialists which enabled them to deliver specialised content. Additionally, the project field facilitators regularly monitored HE-treatment schools to ensure quality and consistency.

### Soap provision strategies

The soap intervention assigned the project field facilitators to regularly distribute six and three small soap bars monthly to SP-treatment schools and randomly chosen ten pupils in each grade in these schools, respectively. No health advice was provided for soap intervention, although a half of the schools received the cross-cutting HE-treatment. Neither schools nor pupils were monitored in terms of how they used the distributed soap bars.

### Project implementation

All HE-treatment schools received all 26 modules of SBHE by the para-teachers, who also instructed the schools to implement a pupil-led cleaning rota. There were a few extra weeks as well as several school health events during which skill-trainings for the first two modules and illness treatment like ORS making were repeated. Due to initial procurement delays, the SP intervention was not implemented as planned, resulting in the distribution of several months’ worth of soap bars at once. Consequently, the intended regular monthly distribution of soap bars was not maintained and instead occurred sporadically.

### Attrition

We collected data from 180 schools at baseline and endline. From baseline to endline, there was 15% attrition (1,087 out of an effective sample of 7,192 children), which was within our sample size calculation set at 20% attrition. More specifically, the attrition rates were 15.0% for both HE-treatment and HE-control groups, while the rates were 15.5%, 12.9%, 14.6%, and 17.1% for the HE-only, SP-only, HESP-treatment, and control schools, respectively. Regressing attrition status on child class, sex, and outcome variables, each interacted with treatment variable, no statistically significant difference was observed between non-treatment and treatment schools. Of those who were in grade 4 at the baseline, i.e., grade 5 during the intervention, endline data were collected from 79.5%; among whom 89.3% had gone to nearby high schools, and the rest stayed in primary schools, repeating the class. Attritted children mostly had gone to other regions. We collected replacement data for 15% or 1,078 pupils for those who attritted at endline. The replacement pupils were of the same sex and the same baseline class. Consequently, the total effective sample size was 7,192 at baseline and 8,991 at endline.

### Outcomes

We had school-level outcome families/groups (denoted here with “S”) as well as child-level outcome families aggregated at the school level (denoted with “C”). The three primary outcome families were (S1) *school hygiene practice & maintenance*, (C1) *child handwashing practice*, and (C2) *child dentalcare practice*. These primary outcomes were considered as the direct indicators of healthy skill-building and behaviour change. *School hygiene practices & maintenance* (S1) consisted of routine latrine cleaning, latrine cleaning days per week, classroom cleaning days per week, classroom rubbish bin provision, latrine brush provision, rubbish treatment methods, ratio of clean-usable latrines to total latrines for each sex, and soap provision for pupils at handwashing place. Routine cleaning practices were self-reported by respondents, typically headteachers, who also accompanied surveyors on inspections of the premises. This approach minimised the likelihood of inaccurate reports, with surveyors verifying cleanliness directly and documenting conditions through photographs. *Handwashing practice* (C1) consisted of handwashing indices^1^ reflecting both handwashing frequency and materials used on each occasion—before eating, after defecation, and after playing—as well as handwashing frequency with soap (0 = *never*; 1 = *sometimes*; 2 = *always*), handwashing frequency using running water, and the handwashing procedure. While these practices were self-reported, surveyors also assessed the thoroughness of handwashing by evaluating the number of correct steps demonstrated by children. Additionally, although it was not included in our primary analysis because data was collected from only 10% of the sample children and measurements were only valid at endline, hand cleanliness was assessed using adenosine triphosphate (ATP) luminometer.^2^ Self-reported *dentalcare practice* (C2) comprised dentalcare frequency in a day (0 = *never*; 1 = *sometimes*; 2 = *always once*; 3 = *always twice or more*) with various material combinations used, that ranged from fingers, branch to brush, with ash, coal, powder and/or paste,^3^ and frequency using brush/branch for daily dentalcare.

Two secondary outcome families were indirect outcomes derived from the primary outcomes. The school-level outcome family was (S2) *schooling*, composed of grade-wise and school-wide attendance rates, defined as the number of students attending school divided by the number of admitted students per grade and per school, respectively. The school-aggregated child outcome family was (C3) *child illnesses*, composed of cough, breathing difficulty, sore throat, fever, running nose and congested nose at present and in the past two-weeks, diarrhoea in the past two-weeks, stomach-ache in the past two-weeks, impetigo, dizziness, fatigue, and appetite loss. While illnesses were self-reported, any external symptoms were verified by the surveyors.

### Statistical analysis

Since the intervention was conducted at school level with children as the primary target, only intent-to-treat (ITT) effect could be measured. The ITT estimates of average treatment effect (ATE) were estimated via the difference-in-differences (DID) estimator adjusting for any pre-existing random differences between the treatment and comparison schools, controlling for time-constant unobservable school and school-aggregated child characteristics. The family-wise mean-standardised average treatment effects were estimated through seemingly unrelated regressions (SUR) that allowed contemporaneous errors to be correlated [24, 25].^4^ This analysis accounted for possible cherry-picking caused by the increased likelihood of finding significant results simply due to conducting many regressions [27, 28]. The DID allowed us not only to estimate the *HE-treatment effect* but also the *period effect* which would reflect the general time trend between baseline and endline, regardless of the treatment status. Note that the fact that some of the outcome variables being binary would not pose a particular challenge in obtaining the average causal effect of a random treatment, as the ATE exhibits differences in probabilities of outcome = 1, as described by [29]. A *school-type* stratification dummy variable was included as a necessary control.

In the DID model, we additionally included a composite spillover/externality index to capture any spillover effects from the HE-treatment schools to other schools. The index was composed of the distances between schools and HE-schools, and the number of pupils attending these schools^5^ (see Additional File S1: A-Fig. S1 for the map of schools). It was not uncommon for pupils from the same neighbourhood to attend different schools, and for them to interact in village/town especially if their schools were in proximity. In such circumstances, the skills and knowledge they had learnt in SBHE sessions might have been talked of. On the other hand, despite advances in communication technology, it was unlikely that guardians would discuss the health skills their children learnt at schools over mobile phones, nor were such skills easily transmittable in this manner.

Finally, the cost-effectiveness analysis was conducted based on the direct labour cost of 18 para-teachers for the project year, the initial training workshop and a refresher workshop, the fixed costs of SBHE digital material development, and the cost of mini-projectors. We provide cost-effectiveness measures in terms of the cost per 0.1 SD increase in the ATE on specific outcome families, dividing the cost per school by the ATE.

## Results

### General baseline observation

Table 2 provides descriptive statistics for the baseline school characteristics and outcome variables categorised in their analytic families for (1) HE-treatment groups (non-HE and HE) and for (2) cross-cutting HESP-treatment groups (control, SP, HE and HESP). While randomisation ideally ensured no statistical difference in school-level variables, it was not possible to completely rule out statistical differences for all aggregated child-level variables. Specifically, two variables concerning handwashing before eating had higher mean values for the non-HE group at 5% statistical significance (indicated in the p-value column in Table 2). While any estimation bias would unlikely favour the HE-treatment group in this case, we dealt with such non-random baseline differences by applying the DID estimator. There were no baseline statistical differences across the four HESP-treatment groups.

**Table 2:** Summary statistics of baseline characteristics and outcome variables by the HE-treatment and the cross-cutting HESP-treatment

	HE-intervention				Cross-cutting HESP-intervention				
	non-HE school	HE school			control school	SP-school	HE-school	HESP-school	
	Mean (SD)	Mean (SD)	p-value ^(a)^		Mean (SD)	Mean (SD)	Mean (SD)	Mean (SD)	p-value ^(b)^
number of schools	90	90			45	45	45	45	
number of surveyed children	3,594	3,598			1,797	1,797	1,799	1,799	
total number of enrolled pupils	232.4 (95.7)	220.8 (97.5)	0.420		229.2 (89.0)	235.6 (102.9)	219.9 (108.3)	221.7 (86.5)	0.860
total number of attended pupils	164.9 (73.3)	159.8 (71.2)	0.640		157.4 (68.0)	172.4 (78.2)	157.4 (77.8)	162.2 (64.7)	0.730
ratio of female pupils enrolled	0.51 (0.04)	0.50 (0.05)	0.525		0.50 (0.04)	0.51 (0.04)	0.50 (0.04)	0.51 (0.05)	0.453
ratio of female pupils attended	0.53 (0.05)	0.53 (0.05)	0.529		0.53 (0.05)	0.53 (0.06)	0.52 (0.04)	0.54 (0.06)	0.357
ratio of female teachers	0.48 (0.31)	0.38 (0.28)	0.026		0.49 (0.31)	0.47 (0.31)	0.37 (0.25)	0.39 (0.31)	0.163
spillover/externality measures^(c)^	0.44 (0.30)	0.30 (0.27)	0.254		0.44 (0.28)	0.43 (0.32)	0.37 (0.22)	0.40 (0.31)	0.674
**Primary Outcomes by Families**									
***S1: school hygiene practice & maintenance***									
routine latrine cleaning	0.37 (0.48)	0.38 (0.49)	0.878		0.38 (0.49)	0.36 (0.48)	0.40 (0.50)	0.36 (0.48)	0.968
* no (%)*	*57 (63.3)*	*56 (62.2)*	*1.000*		*28 (62.2)*	*29 (64.4)*	*27 (60.0)*	*29 (64.4)*	*0.967*
* yes (%)*	*33 (36.7)*	*34 (37.8)*			*17 (37.8)*	*16 (35.6)*	*18 (40.0)*	*16 (35.6)*	
latrine cleaning days/week	0.61 (1.09)	0.66 (1.17)	0.792		0.58 (0.92)	0.64 (1.25)	0.73 (1.27)	0.58 (1.08)	0.903
routine classroom cleaning	0.80 (0.40)	0.72 (0.45)	0.223		0.84 (0.37)	0.76 (0.43)	0.82 (0.39)	0.62 (0.49)	0.059
* no (%)*	*18 (20.0)*	*25 (27.8)*	*0.294*		*7 (15.6)*	*11 (24.4)*	*8 (17.8)*	*17 (37.8)*	*0.060*
* yes (%)*	*72 (80.0)*	*65 (72.2)*			*38 (84.4)*	*34 (75.6)*	*37 (82.2)*	*28 (62.2)*	
classroom cleaning days/week	3.48 (2.49)	2.98 (2.59)	0.188		3.47 (2.48)	3.49 (2.53)	3.24 (2.46)	2.71 (2.71)	0.439
classroom rubbish bin provision	0.29 (0.71)	0.20 (0.60)	0.365		0.31 (0.73)	0.27 (0.69)	0.22 (0.64)	0.18 (0.58)	0.797
* no (%)*	*77 (85.6)*	*81 (90.0)*	*0.495*		*38 (84.4)*	*39 (86.7)*	*40 (88.9)*	*41 (91.1)*	*0.793*
* some (%)*	.	.			.	.	.	.	
* all (%)*	*13 (14.4)*	*9 (10.0)*			*7 (15.6)*	*6 (13.3)*	*5 (11.1)*	*4 ( 8.9)*	
latrine brush provision	0.63 (0.49)	0.61 (0.49)	0.804		0.57 (0.50)	0.69 (0.47)	0.69 (0.47)	0.53 (0.50)	0.294
* no (%)*	*33 (37.1)*	*35 (38.9)*	*0.924*		*19 (43.2)*	*14 (31.1)*	*14 (31.1)*	*21 (46.7)*	*0.290*
* yes (%)*	*56 (62.9)*	*55 (61.1)*			*25 (56.8)*	*31 (68.9)*	*31 (68.9)*	*24 (53.3)*	
rubbish treatment^(d)^	0.40 (0.86)	0.42 (0.86)	0.863		0.40 (0.89)	0.40 (0.84)	0.49 (0.94)	0.36 (0.77)	0.904
* none (%)*	*71 (78.9)*	*70 (77.8)*	*0.867*		*36 (80.0)*	*35 (77.8)*	*34 (75.6)*	*36 (80.0)*	*0.988*
* dump in river/pond (%)*	*7 (7.8)*	*6 ( 6.7)*			*3 ( 6.7)*	*4 ( 8.9)*	*3 ( 6.7)*	*3 ( 6.7)*	
* dump in another land (%)*	*7* *(7.8)*	*10 (11.1)*			*3* *( 6.7)*	*4* *( 8.9)*	*5 (11.1)*	*5 (11.1)*	
* burn/burry in school (%)*	*5 (5.6)*	*4 ( 4.4)*			*3 ( 6.7)*	*2 ( 4.4)*	*3 ( 6.7)*	*1 ( 2.2)*	
clean classrooms	1.19 (0.89)	1.00 (0.91)	0.160		1.07 (0.91)	1.31 (0.85)	0.96 (0.90)	1.04 (0.93)	0.279
* no (%)*	*28 (31.1)*	*37 (41.1)*	*0.358*		*17 (37.8)*	*11 (24.4)*	*19 (42.2)*	*18 (40.0)*	*0.632*
* some (%)*	*17 (18.9)*	*16 (17.8)*			*8 (17.8)*	*9 (20.0)*	*9 (20.0)*	*7 (15.6)*	
* all (%)*	*45 (50.0)*	*37 (41.1)*			*20 (44.4)*	*25 (55.6)*	*17 (37.8)*	*20 (44.4)*	
ratio of clean-usable latrines for boys^(e)^	0.47 (0.48)	0.51 (0.47)	0.567		0.51 (0.47)	0.43 (0.49)	0.49 (0.47)	0.53 (0.47)	0.764
ratio of clean-usable latrines for girls^(e)^	0.49 (0.48)	0.46 (0.47)	0.675		0.52 (0.48)	0.46 (0.49)	0.44 (0.47)	0.48 (0.47)	0.87
soap for pupil at handwashing facility	0.46 (0.80)	0.40 (0.75)	0.630		0.44 (0.78)	0.47 (0.81)	0.47 (0.81)	0.33 (0.67)	0.821
* no (%)*	*66 (73.3)*	*68 (75.6)*	*0.824*		*33 (73.3)*	*33 (73.3)*	*33 (73.3)*	*35 (77.8)*	*0.899*
* yes sometimes (%)*	*7 (7.8)*	*8 (8.9)*			*4 (8.9)*	*3 (6.7)*	*3 ( 6.7)*	*5 (11.1)*	
* yes always (%)*	*17 (18.9)*	*14 (15.6)*			*8 (17.8)*	*9 (20.0)*	*9 (20.0)*	*5 (11.1)*	
***C1: school-aggregated child handwashing practice***									
handwashing index before eating^(f)^	1.35 (1.20)	1.01 (0.98)	0.039		1.38 (1.27)	1.33 (1.13)	0.98 (0.90)	1.05 (1.07)	0.224
handwashing index after defecation^(f)^	5.06 (1.05)	4.90 (0.99)	0.312		5.05 (1.10)	5.06 (1.01)	4.94 (0.97)	4.87 (1.02)	0.77
handwashing index after playing^(f)^	0.64 (0.82)	0.48 (0.59)	0.137		0.71 (0.94)	0.58 (0.68)	0.40 (0.44)	0.57 (0.71)	0.239
handwashing with soap before eating^(g)^	0.19 (0.17)	0.14 (0.14)	0.042		0.20 (0.18)	0.19 (0.16)	0.14 (0.13)	0.15 (0.15)	0.236
handwashing with soap after defecation^(g)^	0.65 (0.17)	0.62 (0.16)	0.327		0.65 (0.18)	0.65 (0.17)	0.63 (0.16)	0.62 (0.17)	0.810
handwashing with soap after playing^(g)^	0.09 (0.12)	0.07 (0.08)	0.14		0.10 (0.13)	0.08 (0.10)	0.06 (0.06)	0.08 (0.10)	0.244
handwashing under running water	0.99 (0.06)	0.97 (0.06)	0.156		0.98 (0.06)	0.99 (0.06)	0.97 (0.07)	0.98 (0.06)	0.440
handwashing procedures^(h)^	1.41 (0.46)	1.38 (0.47)	0.648		1.42 (0.45)	1.40 (0.47)	1.42 (0.49)	1.34 (0.45)	0.849
***C2: school-aggregated child dentalcare practice***									
dentalcare frequency	2.03 (0.13)	2.01 (0.10)	0.234		2.03 (0.14)	2.02 (0.12)	2.00 (0.10)	2.01 (0.10)	0.557
dentalcare materials^(i)^	4.53 (0.62)	4.56 (0.55)	0.798		4.62 (0.61)	4.44 (0.62)	4.46 (0.56)	4.65 (0.52)	0.207
dentalcare with brush/branch	1.49 (0.31)	1.47 (0.24)	0.501		1.54 (0.31)	1.45 (0.30)	1.43 (0.24)	1.50 (0.23)	0.205
**Secondary Outcomes by Families**									
***S2: schooling***									
grade1 attendance rate	0.65 (0.18)	0.69 (0.17)	0.135		0.62 (0.20)	0.68 (0.16)	0.68 (0.17)	0.70 (0.17)	0.154
grade2 attendance rate	0.70 (0.18)	0.72 (0.15)	0.577		0.67 (0.21)	0.73 (0.14)	0.69 (0.16)	0.74 (0.15)	0.127
grade3 attendance rate	0.70 (0.18)	0.69 (0.16)	0.964		0.67 (0.20)	0.73 (0.16)	0.69 (0.15)	0.70 (0.17)	0.411
grade4 attendance rate	0.70 (0.17)	0.71 (0.15)	0.415		0.69 (0.17)	0.71 (0.16)	0.71 (0.15)	0.72 (0.14)	0.790
grade5 attendance rate	0.88 (0.12)	0.88 (0.14)	0.852		0.88 (0.12)	0.87 (0.12)	0.85 (0.16)	0.91 (0.11)	0.323
total attendance rate	0.71 (0.14)	0.72 (0.12)	0.426		0.69 (0.16)	0.73 (0.11)	0.71 (0.12)	0.74 (0.11)	0.261
***C3: school-aggregated child illness***									
cough	0.22 (0.11)	0.23 (0.13)	0.737		0.23 (0.12)	0.22 (0.10)	0.22 (0.13)	0.23 (0.13)	0.93
breathing difficulty	0.01 (0.02)	0.01 (0.03)	0.569		0.01 (0.02)	0.01 (0.02)	0.01 (0.03)	0.02 (0.03)	0.592
sore throat	0.01 (0.01)	0.01 (0.02)	0.403		0.01 (0.02)	0.00 (0.01)	0.01 (0.02)	0.01 (0.02)	0.166
fever	0.07 (0.05)	0.08 (0.07)	0.166		0.07 (0.05)	0.07 (0.05)	0.08 (0.07)	0.08 (0.06)	0.516
running nose	0.31 (0.14)	0.32 (0.14)	0.44		0.30 (0.15)	0.31 (0.14)	0.32 (0.14)	0.32 (0.13)	0.871
congested nose	0.05 (0.06)	0.04 (0.05)	0.492		0.05 (0.06)	0.05 (0.06)	0.04 (0.06)	0.05 (0.05)	0.873
cough in past 2 weeks	0.38 (0.15)	0.41 (0.15)	0.250		0.39 (0.16)	0.38 (0.14)	0.41 (0.14)	0.41 (0.15)	0.720
breathing difficulty in past 2 weeks	0.02 (0.03)	0.03 (0.05)	0.385		0.02 (0.03)	0.02 (0.03)	0.02 (0.04)	0.03 (0.04)	0.238
sore throat in past 2 weeks	0.02 (0.03)	0.02 (0.04)	0.32		0.02 (0.04)	0.02 (0.03)	0.02 (0.04)	0.03 (0.06)	0.433
fever in past 2 weeks	0.25 (0.11)	0.26 (0.13)	0.581		0.23 (0.11)	0.27 (0.11)	0.27 (0.13)	0.25 (0.12)	0.30
running nose in past 2 weeks	0.48 (0.16)	0.51 (0.14)	0.109		0.47 (0.18)	0.48 (0.15)	0.52 (0.13)	0.51 (0.16)	0.422
congested nose in past 2 weeks	0.09 (0.09)	0.10 (0.09)	0.723		0.10 (0.10)	0.08 (0.08)	0.09 (0.08)	0.10 (0.10)	0.581
diarrhoea in past 2 weeks	0.15 (0.06)	0.16 (0.07)	0.586		0.15 (0.06)	0.15 (0.07)	0.17 (0.07)	0.15 (0.07)	0.589
stomach-ache in past 2 weeks	0.36 (0.15)	0.37 (0.14)	0.878		0.40 (0.14)	0.33 (0.15)	0.37 (0.14)	0.36 (0.15)	0.15
impetigo	0.14 (0.09)	0.13 (0.09)	0.362		0.15 (0.08)	0.14 (0.10)	0.12 (0.10)	0.14 (0.09)	0.656
dizziness	0.36 (0.13)	0.35 (0.15)	0.601		0.36 (0.12)	0.35 (0.13)	0.35 (0.16)	0.34 (0.15)	0.842
fatigue	0.33 (0.13)	0.31 (0.16)	0.384		0.34 (0.12)	0.32 (0.14)	0.31 (0.15)	0.31 (0.16)	0.72
appetite loss	0.27 (0.16)	0.27 (0.19)	0.841		0.27 (0.16)	0.27 (0.16)	0.26 (0.20)	0.27 (0.19)	0.97

Notes Text and figures shown in *italics* are dichotomous/ordinal variables and their *frequencies (percentages)*. (a) t-test for numerical variables and chi-square test for categorical variables; (b) analysis of variance test; (c) externality index incorporate distances from all HE-treatment schools as well as the number of attending pupils ($\:1/(J{\cdot}\:\mu\:)\cdot\:{\sum\:}_{k}{lnN}_{k}^{T}\cdot\:ln{N}_{j}\cdot\:{e}^{-{d}_{kj}},$ where *d*_*k*_ is the distance of school *j* from a treatment school *k*, whose effect manifests exponential decay multiplied by the natural logarithm of the total number of attending students in school *j*, *lnN*_*j*_, and that in the treatment school *k*, *lnN*_*k*_, both measured at the baseline, summed up for all *K* treatment schools, and divided by total number of schools *J* times µ, the natural logarithm of the average total attending students of all *J* schools at the baseline, in order to normalize). ; (d) ranging from 0 none, 1 dump in river/pond, 2 dump in another land, 3 burn/burry in school; (e) includes unisex toilets; (f) child handwashing index reflecting frequency and substance usage on each occasion, ranging [0, 6]; (g) child handwashing with soap at each occasion [0, 1]; (h) correct handwashing procedure [0, 8]; (i) dentalcare material combinations (finger; paste; powder; coal; ash; branch; brush) ranging [0, 6]. All child-level outcomes are aggregated and averaged by the school level

In terms of (S1) *school hygiene practice & maintenance*, relatively low value for routine cleaning was noticeable, being less than once a week, although there were high variabilities across schools. Also, low usable latrine availability was pronounced, with both HE-control and HE-treatment schools having only about half of the latrines being clean and usable. In fact, 119 schools (66% of total schools) lacked any unlocked, clean latrines for boys, and 122 schools (68% of total school) lacked any for girls. Among the schools that did have unlocked, clean latrines, the average number of attending boys and girls per latrine was 37 and 41, respectively, taking into account that most grades attended school only for half-a-day. For (C1) *child handwashing practice*, handwashing after defecation had the highest value and handwashing after playing had the lowest value in terms of both frequency and the use of soap. While Bangladeshi people generally ate food with their right hand, handwashing before eating was infrequent. Meanwhile, (C2) *child dentalcare practice* showed reasonably high values, with most children brushing their teeth twice a day. As for (S3) *schooling*, attendance rate varied from 62% for grade 1 to 88% for grade 5, although the mean student number was the highest for grade 1 with 52.7 students and the lowest for grade 5 with 31 students.

### General endline observation

We briefly depict general endline observation of school characteristics and outcome variables across the HE-treatment and cross-cutting HESP-treatment groups as shown in Table 3. These observations are juxtaposed with baseline data in Table 2 to illuminate the progress and impact of the interventions implemented. At endline, the school base characteristics remained similar to the baseline conditions. This stability confirms that any changes in school dynamics or outcomes can be attributed to the interventions rather than shifts in the base characteristics. As for (S1) *school hygiene practice & maintenance*, there was a general improvement from baseline to endline, and particularly among the HE-treatment group. It is noticeable that all schools now reported to have routine classroom cleaning. Many outcome values are statistically different between HE-treatment and non-HE treatment groups. Improvements in rubbish disposal practices were observed, with a decrease in the percentage of schools reporting no waste management strategies and an increase in rubbish bin provision as well as regular waste disposal methods. Endline data also show enhancements in the hygiene infrastructure for both groups. These improvements, also seen in non-HE schools suggest the possibility of school hygiene practices promoted by the interventions having been spread to these non-HE schools. The increased availability of soap at handwashing facilities likely contributed to more frequent and effective handwashing practices among students, a crucial factor in promoting overall health hygiene. Regarding (C1) *child handwashing practice*, there were large improvement across different outcome measures from baseline to endline for both HE-treatment and non-HE treatment schools. Interestingly, handwashing index after defecation did not see much improvement from baseline to endline. There was a small increase in (C2) *dentalcare practices*, with higher engagement in regular brushing and using more appropriate dentalcare materials. These comparative observations for the HE-treatment groups are also reflected in the HESP-treatment groups, depending on the HE-treatment status.

**Table 3:** Summary statistics of endline characteristics and outcome variables by the HE-treatment and the cross-cutting HESP-treatment

	HE-intervention				Cross-cutting HESP-intervention					
	non-HE school	HE school			control school		SP-school	HE-school	HESP-school	
	Mean (SD)	Mean (SD)	p-value ^(a)^		Mean (SD)		Mean (SD)	Mean (SD)	Mean (SD)	p-value ^(b)^
number of schools	90	90			45		45	45	45	
number of surveyed children	4,494	4,497			2,247		2,247	2,250	2,247	
total number of enrolled pupils	229.92 (93.72)	221.98 (92.99)	0.57		225.69 (83.74)		234.16 (103.52)	225.51 (101.89)	218.44 (84.17)	0.89
total number of attended pupils	179.37 (81.59)	173.79 (79.72)	0.64		172.82 (81.19)		185.91 (82.37)	169.20 (84.64)	178.38 (75.16)	0.78
ratio of female pupils enrolled	0.50 (0.04)	0.49 (0.04)	0.23		0.50 (0.04)		0.50 (0.04)	0.49 (0.03)	0.49 (0.05)	0.67
ratio of female pupils attended	0.51 (0.05)	0.51 (0.04)	0.66		0.51 (0.05)		0.51 (0.04)	0.51 (0.04)	0.51 (0.05)	0.88
ratio of female teachers	0.49 (0.30)	0.39 (0.27)	0.03		0.50 (0.31)		0.48 (0.29)	0.35 (0.24)	0.44 (0.29)	0.06
spillover/externality measures^(c)^	0.44 (0.30)	0.39 (0.27)	0.25		0.44 (0.28)		0.43 (0.32)	0.37 (0.22)	0.40 (0.31)	0.67
**Primary Outcomes by Families**										
***S1: school hygiene practice & maintenance***										
routine latrine cleaning	0.61 (0.49)	0.86 (0.35)	< 0.001		0.69 (0.47)		0.53 (0.50)	0.87 (0.34)	0.84 (0.37)	< 0.001
* no (%)*	*35 (38.9%)*	*13 (14.4%)*	*< 0.001*		*14 (31.1%)*		*21 (46.7%)*	*6 (13.3%)*	*7 (15.6%)*	*< 0.001*
* yes (%)*	*55 (61.1%)*	*77 (85.6%)*			*31 (68.9%)*		*24 (53.3%)*	*39 (86.7%)*	*38 (84.4%)*	
latrine cleaning days/week	1.27 (1.42)	1.80 (1.66)	0.02		1.45 (1.58)		1.09 (1.22)	2.15 (1.95)	1.45 (1.24)	0.01
routine classroom cleaning	1.00 (0.00)	1.00 (0.00)	.		1.00 (0.00)		1.00 (0.00)	1.00 (0.00)	1.00 (0.00)	.
* no (%)*	.	.	.		.		.	.	.	.
* yes (%)*	90 (100.0%)	90 (100.0%)			45 (100.0%)		45 (100.0%)	45 (100.0%)	45 (100.0%)	
classroom cleaning days/week	5.25 (1.62)	5.36 (1.55)	0.65		5.38 (1.50)		5.12 (1.75)	5.43 (1.40)	5.28 (1.71)	0.79
classroom rubbish bin provision	0.60 (0.80)	0.94 (0.89)	0.01		0.73 (0.86)		0.47 (0.73)	0.84 (0.88)	1.04 (0.90)	0.01
* no (%)*	54 (60.0%)	38 (42.2%)	0.03		24 (53.3%)		30 (66.7%)	21 (46.7%)	17 (37.8%)	0.10
	18 (20.0%)	19 (21.1%)			9 (20.0%)		9 (20.0%)	10 (22.2%)	9 (20.0%)	
* yes (%)*	18 (20.0%)	33 (36.7%)			12 (26.7%)		6 (13.3%)	14 (31.1%)	19 (42.2%)	
latrine brush provision	0.86 (0.35)	0.97 (0.18)	0.01		0.91 (0.29)		0.80 (0.40)	0.98 (0.15)	0.96 (0.21)	0.02
* no (%)*	13 (14.4%)	3 (3.3%)	0.01		4 (8.9%)		9 (20.0%)	1 (2.2%)	2 (4.4%)	0.02
* yes (%)*	77 (85.6%)	87 (96.7%)			41 (91.1%)		36 (80.0%)	44 (97.8%)	43 (95.6%)	
rubbish treatment^(d)^	1.16 (1.24)	1.48 (1.19)	0.08		1.24 (1.23)		1.07 (1.27)	1.42 (1.22)	1.53 (1.18)	0.29
* none (%)*	43 (47.8%)	24 (26.7%)	0.001		19 (42.2%)		24 (53.3%)	13 (28.9%)	11 (24.4%)	0.03
* dump in river/pond (%)*	10 (11.1%)	27 (30.0%)			6 (13.3%)		4 (8.9%)	14 (31.1%)	13 (28.9%)	
* dump in another land (%)*	17 (18.9%)	11 (12.2%)			10 (22.2%)		7 (15.6%)	4 (8.9%)	7 (15.6%)	
* burn/burry in school (%)*	20 (22.2%)	28 (31.1%)			10 (22.2%)		10 (22.2%)	14 (31.1%)	14 (31.1%)	
clean classrooms	1.60 (0.65)	1.67 (0.67)	0.50		1.58 (0.58)		1.62 (0.72)	1.53 (0.76)	1.80 (0.55)	0.24
* no (%)*	8 (8.9%)	10 (11.1%)	0.13		2 (4.4%)		6 (13.3%)	7 (15.6%)	3 (6.7%)	0.01
* some (%)*	20 (22.2%)	10 (11.1%)			15 (33.3%)		5 (11.1%)	7 (15.6%)	3 (6.7%)	
* all (%)*	62 (68.9%)	70 (77.8%)			28 (62.2%)		34 (75.6%)	31 (68.9%)	39 (86.7%)	
ratio of clean-usable latrines for boys^(e)^	0.83 (0.37)	0.88 (0.33)	0.39		0.81 (0.39)		0.86 (0.35)	0.91 (0.29)	0.84 (0.37)	0.59
ratio of clean-usable latrines for girls^(e)^	0.78 (0.41)	0.86 (0.35)	0.21		0.76 (0.43)		0.81 (0.39)	0.96 (0.21)	0.76 (0.43)	0.04
soap for pupil at handwashing facility	1.10 (0.69)	1.43 (0.64)	< 0.001		1.04 (0.64)		1.16 (0.74)	1.49 (0.66)	1.38 (0.61)	0.01
* no (%)*	17 (18.9%)	7 (7.8%)	0.004		8 (17.8%)		9 (20.0%)	4 (8.9%)	3 (6.7%)	0.02
* yes sometimes (%)*	47 (52.2%)	37 (41.1%)			27 (60.0%)		20 (44.4%)	15 (33.3%)	22 (48.9%)	
* yes always (%)*	26 (28.9%)	46 (51.1%)			10 (22.2%)		16 (35.6%)	26 (57.8%)	20 (44.4%)	
***C1: school-aggregated child handwashing practice***										
handwashing index before eating^(f)^	4.03 (0.53)	4.21 (0.58)	0.02		4.00 (0.51)		4.05 (0.55)	4.10 (0.55)	4.32 (0.60)	0.03
handwashing index after defecation^(f)^	5.11 (0.25)	5.18 (0.24)	0.07		5.16 (0.28)		5.07 (0.22)	5.14 (0.24)	5.22 (0.25)	0.05
handwashing index after playing^(f)^	3.37 (0.62)	3.45 (0.57)	0.33		3.45 (0.64)		3.28 (0.59)	3.47 (0.63)	3.43 (0.51)	0.40
handwashing with soap before eating^(g)^	0.35 (0.18)	0.41 (0.20)	0.02		0.34 (0.17)		0.36 (0.19)	0.37 (0.19)	0.45 (0.20)	0.03
handwashing with soap after defecation^(g)^	0.89 (0.09)	0.92 (0.08)	0.02		0.89 (0.08)		0.89 (0.10)	0.91 (0.10)	0.94 (0.06)	0.04
handwashing with soap after playing^(g)^	0.25 (0.15)	0.28 (0.15)	0.16		0.27 (0.18)		0.24 (0.13)	0.27 (0.17)	0.30 (0.14)	0.36
handwashing under running water	1.90 (0.09)	1.91 (0.11)	0.41		1.91 (0.07)		1.89 (0.10)	1.91 (0.11)	1.92 (0.11)	0.45
handwashing procedures^(h)^	2.04 (0.37)	2.81 (0.57)	< 0.001		2.09 (0.35)		1.99 (0.37)	2.84 (0.63)	2.78 (0.51)	< 0.001
***C2: school-aggregated child dentalcare practice***										
dentalcare frequency	2.11 (0.14)	2.18 (0.20)	0.01		2.16 (0.17)		2.07 (0.09)	2.17 (0.20)	2.19 (0.19)	0.00
dentalcare materials^(i)^	5.34 (0.33)	5.50 (0.27)	< 0.001		5.39 (0.32)		5.29 (0.33)	5.53 (0.25)	5.47 (0.29)	< 0.001
dentalcare with brush/branch	1.94 (0.20)	2.07 (0.22)	< 0.001		2.00 (0.20)		1.89 (0.18)	2.07 (0.22)	2.07 (0.23)	< 0.001
**Secondary Outcomes by Families**										
***S2: schooling***										
grade1 attendance rate	0.78 (0.15)	0.77 (0.17)	0.82		0.77 (0.13)		0.78 (0.18)	0.73 (0.19)	0.81 (0.13)	0.15
grade2 attendance rate	0.72 (0.20)	0.74 (0.20)	0.66		0.68 (0.23)		0.77 (0.16)	0.71 (0.22)	0.77 (0.18)	0.08
grade3 attendance rate	0.77 (0.16)	0.78 (0.15)	0.71		0.74 (0.16)		0.80 (0.15)	0.75 (0.16)	0.81 (0.14)	0.05
grade4 attendance rate	0.79 (0.16)	0.80 (0.15)	0.81		0.77 (0.19)		0.81 (0.11)	0.76 (0.17)	0.83 (0.12)	0.10
grade5 attendance rate	0.87 (0.11)	0.88 (0.15)	0.71		0.86 (0.12)		0.89 (0.10)	0.87 (0.17)	0.89 (0.13)	0.67
total attendance rate	0.78 (0.12)	0.78 (0.13)	0.85		0.76 (0.13)		0.80 (0.11)	0.75 (0.15)	0.81 (0.11)	0.04
***C3: school-aggregated child illnesses***										
cough	0.14 (0.08)	0.12 (0.06)	0.20			0.14 (0.08)	0.14 (0.08)	0.12 (0.06)	0.13 (0.07)	0.61
breathing difficulty	0.02 (0.03)	0.02 (0.02)	0.13			0.02 (0.02)	0.03 (0.03)	0.02 (0.02)	0.02 (0.03)	0.27
sore throat	0.01 (0.01)	0.01 (0.02)	0.35			0.01 (0.02)	0.00 (0.01)	0.01 (0.01)	0.01 (0.02)	0.24
fever	0.06 (0.05)	0.06 (0.05)	0.47			0.06 (0.05)	0.06 (0.05)	0.06 (0.04)	0.06 (0.05)	0.76
running nose	0.19 (0.08)	0.19 (0.08)	0.49			0.19 (0.08)	0.20 (0.09)	0.19 (0.08)	0.19 (0.08)	0.73
congested nose	0.06 (0.05)	0.06 (0.04)	0.87			0.05 (0.06)	0.06 (0.05)	0.06 (0.04)	0.05 (0.04)	0.95
cough in past 2 weeks	0.28 (0.12)	0.28 (0.11)	0.60			0.27 (0.11)	0.30 (0.12)	0.27 (0.10)	0.28 (0.11)	0.52
breathing difficulty in past 2 weeks	0.06 (0.06)	0.05 (0.05)	0.38			0.05 (0.05)	0.07 (0.06)	0.05 (0.04)	0.05 (0.05)	0.29
sore throat in past 2 weeks	0.02 (0.03)	0.02 (0.02)	0.20			0.02 (0.03)	0.02 (0.03)	0.02 (0.02)	0.02 (0.02)	0.51
fever in past 2 weeks	0.22 (0.09)	0.22 (0.08)	0.89			0.21 (0.10)	0.23 (0.09)	0.21 (0.08)	0.22 (0.08)	0.66
running nose in past 2 weeks	0.36 (0.11)	0.36 (0.08)	0.76			0.34 (0.11)	0.38 (0.11)	0.36 (0.08)	0.36 (0.09)	0.37
congested nose in past 2 weeks	0.12 (0.07)	0.12 (0.07)	0.93			0.11 (0.07)	0.13 (0.07)	0.12 (0.07)	0.12 (0.07)	0.76
diarrhoea in past 2 weeks	0.12 (0.07)	0.11 (0.06)	0.49			0.11 (0.07)	0.13 (0.07)	0.10 (0.06)	0.12 (0.06)	0.13
stomach-ache in past 2 weeks	0.31 (0.11)	0.32 (0.10)	0.32			0.33 (0.13)	0.29 (0.10)	0.33 (0.10)	0.32 (0.11)	0.23
impetigo	0.06 (0.05)	0.06 (0.04)	0.68			0.06 (0.05)	0.07 (0.05)	0.06 (0.04)	0.06 (0.04)	0.74
dizziness	0.22 (0.10)	0.23 (0.09)	0.72			0.22 (0.09)	0.22 (0.11)	0.21 (0.10)	0.24 (0.08)	0.52
fatigue	0.21 (0.10)	0.20 (0.09)	0.50			0.22 (0.10)	0.19 (0.08)	0.19 (0.09)	0.21 (0.08)	0.32
appetite loss	0.24 (0.10)	0.25 (0.11)	0.75			0.25 (0.10)	0.23 (0.11)	0.23 (0.10)	0.27 (0.11)	0.26

Notes Text and figures shown in *italics* are dichotomous/ordinal variables and their *frequencies *(*percentages*). (a) t-test for numerical variables and chi-square test for categorical variables; (b) analysis of variance test; (c) externality index incorporate distances from all HE-treatment schools as well as the number of attending pupils ($\:1/(J\cdot\:\mu\:)\cdot\:{\sum\:}_{k}{lnN}_{k}^{T}\cdot\:ln{N}_{j}\cdot\:{e}^{-{d}_{kj}},$ where *d*_*k*_ is the distance of school *j* from a treatment school *k*, whose effect manifests exponential decay multiplied by the natural logarithm of the total number of attending students in school *j*, *lnN*_*j*_, and that in the treatment school *k*, *lnN*_*k*_, both measured at the baseline, summed up for all *K* treatment schools, and divided by total number of schools *J* times µ, the natural logarithm of the average total attending students of all *J* schools at the baseline, in order to normalize). ; (d) ranging from 0 none, 1 dump in river/pond, 2 dump in another land, 3 burn/burry in school; (e) includes unisex toilets; (f) child handwashing index reflecting frequency and substance usage on each occasion, ranging [0, 6]; (g) child handwashing with soap at each occasion [0, 1]; (h) correct handwashing procedure [0, 8]; (i) dentalcare material combinations (finger; paste; powder; coal; ash; branch; brush) ranging [0, 6]. All child-level outcomes are aggregated and averaged by the school level

**Fig. 2:**
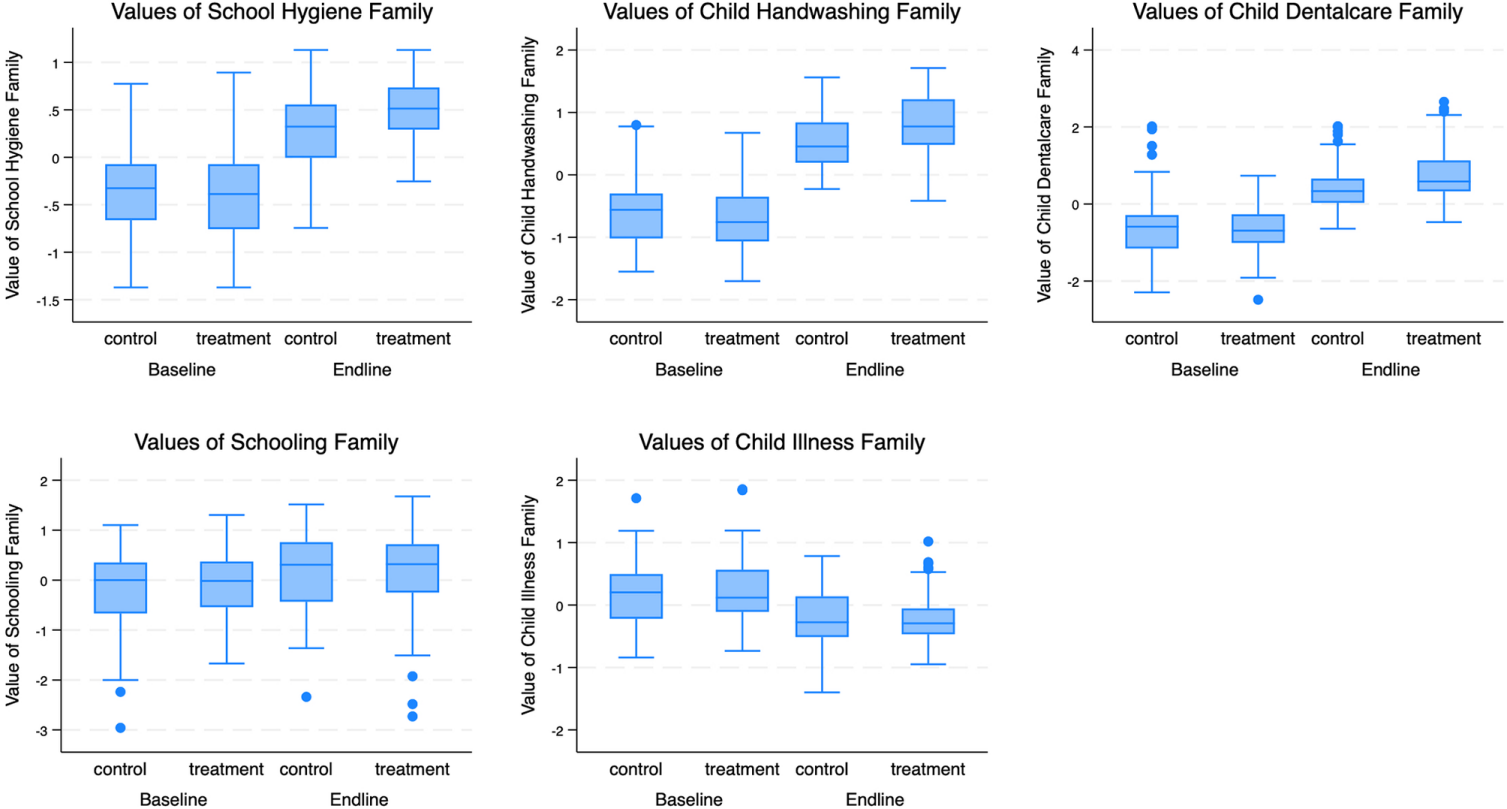
Boxplot Comparison of Outcome Family-Index Values by HE-treatment Groups at Baseline and Endline. The boxplot displays the distribution of family-wise index mean values across two treatment groups (“control” and “treatment”) and two time points (“Baseline” and “Endline”) for each of five outcome families. Each boxplot segment represents the interquartile range (IQR) of the index values, with the median marked by a horizontal line within each box. The whiskers extend to the most extreme data points that are no more than 1.5 times the IQR from the box, and outliers are depicted as individual points beyond the whiskers

As a brief note on the secondary outcomes, (S2) *schooling* in terms of attendance rate slightly improved across grades except for grade 5, but did not significantly change from baseline to endline. In terms of (C3) *child illnesses*, there were no significant differences between treatment groups, and there were mixed findings for cold-related symptoms comparing baseline and endline. Nonetheless, the reduction in other symptoms, i.e., impetigo, dizziness, fatigue, and appetite loss at endline across treatment groups was pronounced. For the ATP measures which were not included in the analysis due to their sample size and its availability only at endline, descriptive statistics of the measures aggregated at the school level are provided in Additional File S2: A-Table S1. Although no statistically significant difference was observed between the HE-treatment and non-HE treatment groups, likely due to large standard errors and a small sample size, the improvement rate was nearly three-fold for HE-treatment group compared to non-HE-treatment group. The aggregated family outcome differences between HE-treatment and non-HE treatment schools at baseline and endline are depicted in Fig. 2.

### Treatment, period and spillover effects

All the analysis were conducted at school level. Table 4 gives the average-effect estimates for each of family-wise outcome for the HE-intervention. All primary outcomes related to hygiene practices had positive HE-treatment effects with statistical significance at the 1% level. The effects of HE-treatment resulted on average in 0.32 higher standard deviation (SD) [0.14–0.49] (*p* < 0.001) of *school hygiene practice & maintenance* than control schools. Likewise, children in the HE-treatment schools on average had better *(school-aggregated) child handwashing practice* by 0.47 SD [0.26–0.67] (*p* < 0.001), and better *child dentalcare practice* by 0.43 SD [0.17–0.70] (*p* < 0.01). In addition to the HE-treatment effects, both period and spillover exerted positive effects on these hygiene practice outcomes. The period effects were pronounced with high magnitude of impact at 0.60 SD [0.47–0.72] (*p* < 0.001) higher *school hygiene practice & maintenance*, 1.18 SD [1.04–1.32] (*p* < 0.001) higher *child handwashing practice*, and 1.06 SD [0.87–1.24] (*p* < 0.001) higher* child dentalcare practice*. Spillover effects were estimated to lead to 0.27 SD [0.11–0.42] (*p* < 0.001) increase in *school hygiene practice & maintenance*, 0.41 SD [0.18–0.64] (*p* < 0.001) increase in *child handwashing practice*, and 0.70 SD [0.41–0.99] (*p* < 0.001) increase in *child dentalcare practice*. While no statistically significant HE-treatment effects were found among the secondary outcomes, period had positive effects on both *schooling* by 0.38 SD [0.16–0.60] (*p* < 0.001) and child illness reduction by -0.36 SD [-0.51–-0.22] (*p* < 0.001) (negative AES-coefficient signifying positive effects), indicating improvements in these outcomes over time. No spillover effects were detected for these secondary outcomes.

**Table 4:** Estimated average effect size (AES) of HE-treatment effects, period effects and spillover effects on five school-level outcome families (*N* = 360)

	primary outcomes					secondary outcomes			
	(S1) school hygiene practice & maintenance	(C1) school-aggregated child handwashing		(C2) school-aggregated child dentalcare			(S2) schooling	(C3) school-aggregated child illnesses	
	AES-coefficient [95%CI]		AES-coefficient [95%CI]		AES-coefficient [95%CI]		AES-coefficient [95%CI]		AES-coefficient [95%CI]
HE-treatment	0.32*** [0.14–0.49]		0.47*** [0.26–0.67]		0.43**[0.17–0.70]		-0.06 [-0.36–0.25]		-0.10 [-0.30–0.10]
period	0.61*** [0.49–0.74]		1.18*** [1.04–1.32]		1.06*** [0.87–1.24]		0.38*** [0.16–0.60]		-0.36*** [-0. 51–-0.22]
spillover	0.26*** [0.11–0.41]		0. 41*** [0.18–0.64]		0.70*** [0.41–0.99]		0.11 [-0.21–0. 42]		-0.13 [-0.31–0.04]

Notes Each column represents a separate regression on a family of outcomes estimated by seemingly unrelated regressions (SUR). AES-coefficient is the mean standardized average effect size from SUR applying the difference-in-differences (DID) estimator that controls for HE-group and school type. Outcome family compositions are: (S1) *school hygiene practice &* *maintenance*: latrine cleaning rota, latrine cleaning days per week, classroom cleaning days per week, classroom rubbish bin provision, latrine brush provision, rubbish disposal method, clean classrooms, ratio of clean-usable latrine for boys, ratio of clean-usable latrine for girls, soap provision for pupils at handwashing facility; (C1) *school-aggregated child handwashing practice*: handwashing habits (washing frequency in each occasion (before eating, after defecation, after playing); used substances (soap, ash, mud or water only); washing with soap in each occasion; wash with running water); (C2) *school-aggregated deltalcare* *practice*: dentalcare frequency, dentalcare materials, frequency of brush/branch use; (S2) *schooling*: attendance rate for each grade1 ~ 5, school-wide attendance rate; (C3) *school-aggregated* *child illnesses*: cold symptoms at present and in the past two-weeks, i.e., cough, breathing difficulty, sore throat, fever, running nose, congested nose; diarrhoea in two-weeks; stomach-ache in two-weeks; dizziness; fatigue; appetite loss. Significance level: + *p* < 0.1, * *p* < 0.05, ** *p* < 0.01, ***p < 0.001; 95% confidence interval (CI) in brackets. 360 samples are 90 HE-schools and 90 control-schools in the baseline and endline

Table 5 provides the average-effect estimates for each of family-wise outcome for the cross-cutting HESP-intervention groups, HE, SP and HESP. The HE-only-treatment was found to result in higher *school hygiene practice & maintenance* by an average effect of 0.23 SD [-0.01–0.48] (*p* < 0.10), higher *child handwashing* by an average effect of 0.31 SD [0.03–0.59] (*p* < 0.05), and higher *child dentalcare* by an average effect of 0.44 SD [0.08–0.80] (*p* < 0.05). The HESP-treatment had additional effect on *school hygiene practice & maintenance* by an average of 0.31 SD [0.06–0.57] and on *child handwashing* by an average of 0.48 SD [0.21–0.76], while it had no effect on other outcomes. The SP-only-treatment had no statistically significant effect on any outcomes. The period effect was statistically significant across all primary and secondary outcomes, showing a magnitude similar to that observed in the HE-treatment analysis. Spillover effects likewise exhibited similar results to those in the HE-treatment analysis. No harm was observed in any group.

**Table 5:** Estimated average effect size (AES) of HESP-treatment effect, period effect and spillover effect on five school-level outcome families (*N* = 360)

	primary outcomes					secondary outcomes			
	(S1) school hygiene practice & maintenance		(C1) school-aggregated child handwashing		(C2) school-aggregated child dentalcare		(S2) schooling		(C3) school-aggregated child illnesses
	AES-coefficient [95%CI]		AES-coefficient [95%CI]		AES-coefficient [95%CI]		AES-coefficient [95%CI]		AES-coefficient [95%CI]
SP-only-treatment	-0.09 [-0.33–0.16]		-0.12 [-0.39–0.16]		-0.1 [-0.44–0.24]		0.05 [-0.35–0.45]		0.09 [-0.19–0.38]
HE-only-treatment	0.23+ [-0.01–0.48]		0.31* [0.03–0.59]		0.44* [0.08–0.80]		-0.1 [-0.54–0.33]		-0.08 [-0.35–0.19]
HESP-treatment	0.31* [0.06–0.57]		0.48*** [0.21–0.76]		0.27 [-0.09–0.62]		0.05 [-0.35–0.44]		-0.02 [-0.31–0.26]
period	0.66** [0.49–0.84]		1.20*** [1.00–1.40]		1.09*** [0.83–1.34]		0.33* [0.02–0.64]		-0.41*** [-0. 61–-0.21]
spillover	0.27*** [0.12–0.42]		0.40*** [0.18–0.62]		0.66*** [0.39–0.93]		0.09 [-0.20–0.38]		-0.13 [-0.30–0.04]

Note: Each column represents a separate regression on a family of outcomes estimated by seemingly unrelated regressions (SUR). AES-coefficient is the mean standardised average effect size from SUR applying the difference-in-differences (DID) estimator controls for each of four HESP-groups and school type. Outcome family compositions are: (S1) *school hygiene practice &* *maintenance*: latrine cleaning rota, latrine cleaning days per week, classroom cleaning days per week, classroom rubbish bin provision, latrine brush provision, rubbish disposal method, clean classrooms, ratio of clean-usable latrine for boys, ratio of clean-usable latrine for girls, soap provision for pupils at handwashing facility; (C1) *school-aggregated* *child* *handwashing practice*: handwashing habits (washing frequency in each occasion (before eating, after defecation, after playing); used substances (soap, ash, mud or water only); washing with soap in each occasion; wash with running water); (C2) *schoo*l-*aggregated child dentalcare practice*: dental care frequency, dentalcare materials, frequency of brush/branch use; (S2) *schooling*: attendance rate for each grade1 ~ 5, school-wide attendance rate; (C3) *school*-*aggregated* *child illnesses*: cold symptoms at present and in the past two-weeks, i.e., cough, breathing difficulty, sore throat, fever, running nose, congested nose; diarrhoea in two-weeks; stomach-ache in two-weeks; dizziness; fatigue; appetite loss. Significance level: + *p* < 0.1, * *p* < 0.05, ** *p* < 0.01, ***p < 0.001; 95% confidence interval (CI) in brackets. 360 samples are 45 HE-schools, 45 HESP-schools, 45 SP-schools and 45 control-schools in the baseline and endline

### Cost-effectiveness of SBHE

The direct labour cost of 18 para-teachers for the project year was 14,466 USD. In addition to this, the project incurred the initial training workshop and refreshers workshop, amounting to 1,156 USD. Apart from these running costs (totalling 15,622 USD), there were fixed costs of SBHE material development (4,943 USD) and of 23 mini-projectors (including five extra ones) (3,460 USD). Since the HE materials were in digital form, they could be used almost perpetually in principle. However, considering that materials could get outdated, we conservatively assumed its lifetime as 10 years without major revision. As for mini-projectors, we assumed that such equipment would depreciate in 5 years. Supposing an equal use value in each year, the annual costs of materials and projectors were 494 USD and 346 USD, respectively. This made the total annual cost of SBHE with fixed cost 16,462 USD for the 90 HE-schools. Thus, per school annual running cost was 160.73 USD excluding the workshops or 182.91 USD including the workshops and other fixed cost. The number of admitted pupils or attended pupils in HE schools at the time of endline, thus assumed to have been treated, were 19,978 or 15,641 (those in non-HE schools were 20,693 and 16,143, respectively). This made the annual cost of para-teachers and the annual total cost of SBHE per admitted student at 0.72 USD and 0.82 USD, respectively, and these costs per attended student at 0.92 USD and 1.05 USD, respectively.

In terms of total cost-effectiveness, the cost per 0.1 SD improvement^6^ was 57.2 USD (=182.91/3.2) for *school hygiene practice*, 38.9 USD (=182.91/4.7) for *child handwashing*, and 42.5 USD (=182.91/4.3) for *child dentalcare* per school. Thes achieved effects concern all included outcome variables which were mean-standardised. Divided by the average attendance of 173.8 students in an HE-school at endline, these costs amounted to approximately 0.2 ~ 0.3 USD per pupil across these outcome families. Adding the spillover effects, the USD per of school-wise 0.1 SD improvement amounted to 31.5 USD (=182.91/(3.2 + 2.6)) for *school hygiene practice*, 20.8 USD (=182.91/(4.7 + 4.1)) for *child handwashing*, and 16.2 USD (=182.91/(4.3 + 7.0)) for *child dentalcare.*

## Discussion

Our study demonstrated positive impacts of SBHE on all primary outcomes, school hygiene practice, child handwashing and child dentalcare. The Difference-in-Differences (DID) approach allowed us to isolate the effect of the intervention by comparing changes over time between the groups, adjusting for common trends and time-invariant differences, thus offering a clearer understanding of the intervention’s impact.

While outcomes such as handwashing habits were based on self-reports by the children, which could pose a limitation to our findings, the demonstration of correct handwashing procedures evaluated by the surveyors lends credibility to the reported behaviours. Furthermore, the fact that children did not uniformly report ‘correct practice’ on all recommended occasions buttresses the objectivity of their responses. Although not included in this study, the objective evidence from adenosine triphosphate (ATP) measurement assessing hand cleanliness provided some evidence of improved practice [30]. The handwashing index after defecation showed only a marginal improvement from baseline across treatment groups, indicating that any bias due to over-reporting would have likely been symmetrical across both groups, thus mitigating its impact on the differential treatment effect estimation. The positive HE effects on dentalcare resonated with those of the successful SBHE studies conducted by [11, 31].

The additional soap provision intervention did not yield positive effect on its own. However, when combined with SBHE (i.e., HESP-treatment), it enhanced hygiene-related outcomes, indicating a synergistic effect. The lower statistical significance of the HE-only-treatment in the cross-cutting analysis may be attributed to insufficient statistical power. Thus, although we cannot be definite, it remains sceptical if soap provision had any additional impact on SBHE. It should also be reiterated that soap provision did not involve any instruction, and we did not monitor how the distributed soap bars were used.

There are several plausible reasons for limited findings for secondary outcomes of schooling and child health (illnesses). Either a weekly SBHE was insufficient or the data lacked statistical power due to the actual effect size, sample size, and/or contamination by the spillover and period effects.^7^ Although the inclusion of spillover/externality measures did not affect the effect size of other estimated coefficients, such spillover effects might have diluted the treatment effect estimate itself. As we have seen, there was a large general improvement from baseline to endline across treatment groups for all the outcomes, including non-cold-related child illnesses. Another reason for no treatment effect on child illnesses could be due to the attrition of relatively unhealthy children, causing a downward bias for the estimated effects. Additionally, there could have been some cancelling-off effect between SBHE sessions and PE sessions, as SBHE sessions were carried out using PE class allotment. If PE classes, or playing around, were assumed to have positive health effects, estimated HE effects could be biased toward zero.

The strengths of our study include the effective addressing of common barriers in health education [4, 15, 16, 32] by employing para-teachers and using mobile projectors, which enhanced engagement and reduced costs. By estimating the mean-standardised average treatment effect on the families of outcomes, we avoided over-identification of statistically significant results. Another strength was the explicit measurement of spillover effects of SBHE. While spillover effects are generally considered as nuisance to the RCT evaluation, in actuality, they could disseminate part of SBHE without extra cost to other schools and pupils, thereby reducing the de facto cost of the intervention. The cost-effectiveness analysis demonstrated that improvements in healthy behaviours were achieved at a low cost per pupil., ranging from 0.2 ~ 0.3 USD per 0.1 SD change in outcome measures per pupil per annum, and the cost was even less further accounting for spillover effects. Although not directly comparable due to differences in measurement units, these figures could be significantly lower than those reported in the above-cited studies [4, 7].

## Conclusions

The evidence we presented indicated the establishment of new healthy practices via SBHE, added by the period and spillover effects to other non-HE schools. Once acquired, healthy habits are financially non-costly and sustainable per se, and they can reinforce the positive impacts of other supply-based intervention such as hygiene infrastructure construction and nutrition provision. Cleaner and better maintained school infrastructure and self-hygiene, as sustained by the evidence, were also expected to serve as infection prevention from diseases and parasites. With the number of schools and children involved int the study, the evidence of acquired healthy habits and spillover effects is suggestive of the applicability of SBHE to other schools and children in resource-poor settings. Although our study was for a limited period, further research looking into how well such healthy environment and behaviour sustain to become a norm and how such healthy habits leads to improved health would be warranted. Along with economic development, healthy behavioural changes and norms are expected to contribute to breaking the vicious circle of ill health, poor education and poverty, and sustainable health improvement.

## Electronic supplementary material

Below is the link to the electronic supplementary material.

Supplementary Material 1

Supplementary Material 2

## Data Availability

The datasets used and/or analysed during the current study are available from the corresponding author on reasonable request.

## Footnotes

1 Child-level handwashing indices are the summation of three indices for each of the following occasions: before eating; after defecation; after playing. The score is given according to the handwashing frequency with different material combinations: 0 = *do not wash*; 1 = *sometime wash with mud (no soap/water/ash)*; 2 = *always wash with mud (no soap/water/ash)*; 3 = *sometime wash with ash (no soap/water)*; 4 = *always wash with ash (no soap/water)*; 5 = *sometime wash with water (no soap)*; 6 = *always wash with water (no soap)*; 7 = *sometime wash with soap*; 8 = *always wash with soap*. While the usage of soap is considered more hygienic, rubbing hands with ash/mud/soil is considered to be effective in removing faecal coliforms, although the risk of contamination by microbial pathogens, helminths, and chemical toxins is present [21, 22]. In rural Bangladesh, washing hands with only water before eating/cooking, or with ash/mud/soil after defecation was widely seen. All child-level outcomes were aggregated at school level in this analysis.

2 This device detects actively growing microorganisms measuring the light emitted from the reaction of ATP with the firefly enzyme luciferase. Although smaller figures suggest cleaner hands, i.e., less microorganisms, there are large individual variations in the figures. Thus, tests were conducted before and after handwashing to measure whether appropriate handwashing had been done. The ATP improvement rate was calculated as (ATP-before – ATP-after)/ATP-before, making the larger the figure the higher the improvement. Only the endline ATP was valid due to ill measurement method applied at baseline. School-aggregated descriptive statics are given in Additional File S2 A-Table 1. See Omura et al. (20024) [23] or its earlier version by Omura (2024) [30] for the child-level analysis using ATP measure as well as further child-level analysis.

3 Child-level dentalcare materials are given the following scores: 0 = *not applicable/finger*; 1 = *paste*,* powder*,* coal*,* ash*; 2 = *branch only*; 3 = *brush only*; 4 = *branch + paste/powder/coal/ash*; 5 = *brush & branch + paste/powder/coal/ ash*; 6 = *brush + paste/powder/coal/ash*; 7 = *brush + paste only*.

4 For treatment effect *β*_*j*_ for each outcome *y*_*j*_, the average effect size (AES) is expressed as $\:\tau\:\:=\:\frac{1}{J}{\sum\:}_{j=1}^{J}\frac{{\varvec{\beta\:}}_{\varvec{j}}}{{\sigma\:}_{j}}$ where *σ*_*j*_ is the standard deviation of outcome *y*_*j*_ in the comparison group [25, 26].

5 The spillover index takes the following form: $\:\frac{1}{J\cdot\:\mu\:}\cdot\:{\sum\:}_{k}{lnN}_{k}^{T}\cdot\:ln{N}_{j}\cdot\:{e}^{-{d}_{kj}},$ where *d*_*k*_ is the distance of school *j* from a treatment school *k*, whose effect manifests exponential decay – the further the distance, the quicker the effects decay – multiplied by the natural logarithm of the total number of attending students in school *j*, *lnN*_*j*_, and that in the treatment school *k*, *lnN*_*k*_, both measured at baseline, summed up for all *K* treatment schools, and divided by total number of schools *J* times *µ*, the natural logarithm of the average total attending students of all *J* schools at baseline, in order to normalise. Distances were calculated using global positioning system data of each school location embedded in photos taken during the survey. For *e*^*− d*^, the closer the schools, the more the figure is larger and thus the spillover effects, e.g., for 500 m it is 0.606, while for 3 km and 6 km, it is 0.050 and 0.002, respectively. We used baseline attending students’ data rather than admitted students’ data because the latter could be significantly different from the actual number. It was a routine practice especially for non-government primary schools (RNGPS) to inflate the number of admitted students in order to obtain more school funds (a pairwise correlation between admitted and attended pupils for government primary schools (GPS) and RNGPS at baseline were 0.933 and 0.819, respectively). It requires a log form because the rate of increase in the number of contacts is not proportional to the rate of increase in the number of pupils, due to an increased probability of contacting the same person.

6 As noted earlier, the effect size was assumed to be 0.15 for sample size calculation. In this context, the cost-effectiveness calculation based on 0.1 SD improvement across all outcomes are considered appropriate.

7 Regarding the cross-cutting treatment analysis, despite this intervention deign having the advantage of efficient use of samples, it likely suffered from insufficient statistical power, as it reduced the sample size to 45 per treatment, and the DID estimator amounted to including three additional controls for treatment groups reducing the degrees of freedom. The presumed effect size and sampling number calculation might not have been sufficient especially for the secondary outcomes – although 180 schools were maximum budget-wise.
