# Supplementary material for: Promoting healthy practices among schools and children in rural bangladesh: a randomised controlled trial of skill-based health education
Source: BMC Public Health. 2024 Nov 27;24:3300. doi: 10.1186/s12889-024-20787-0 (PMC11600675; doi:10.1186/s12889-024-20787-0)
Supplement: Supplementary file 1 — Supplementary Material 1 [file 12889_2024_20787_MOESM1_ESM.pdf]

A-Figure 1 Project School Map in Jhenaidah, Bangladesh

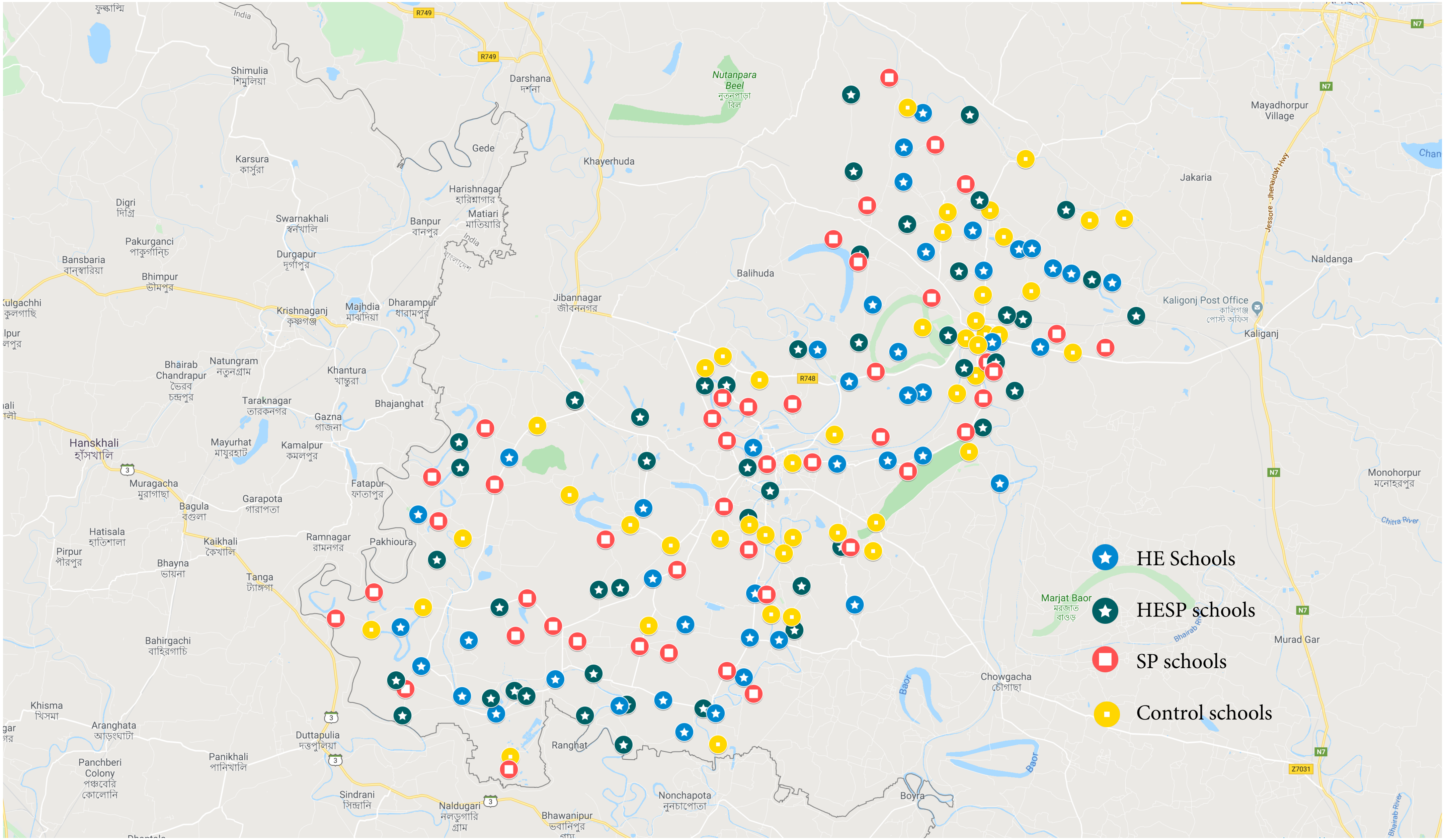

Note: Map created using Google map.

Map data ©2019 2 km
