# Supplementary material for: Promoting healthy practices among schools and children in rural bangladesh: a randomised controlled trial of skill-based health education
Source: BMC Public Health. 2024 Nov 27;24:3300. doi: 10.1186/s12889-024-20787-0 (PMC11600675; doi:10.1186/s12889-024-20787-0)
Supplement: Supplementary file 2 — Supplementary Material 2 [file 12889_2024_20787_MOESM2_ESM.pdf]

A-Table 1 School-Averaged Adenosine Triphosphate (ATP) Measures of Hand Cleanliness at Endline (N =180)

	HE-intervention			Cross-cutting HESP-intervention				
	**non-HE school**	**HE school**		**control school**	**SP-school**	**HE-school**	**HESP-school**	
	Mean (SD)	Mean (SD)	p-value (a)	Mean (SD)	Mean (SD)	Mean (SD)	Mean (SD)	p-value (b)
N	90 (50.0%)	90 (50.0%)		45 (25.0%)	45 (25.0%)	45 (25.0%)	45 (25.0%)	
ATP before handwashing	3006.65 (1079.99)	2930.84 (1022.77)	0.629	3049.63 (1179.87)	2963.66 (981.51)	2980.30 (1022.31)	2881.38 (1032.35)	0.901
ATP after handwashing	1863.10 (931.27)	1741.57 (825.15)	0.355	2041.58 (1044.63)	1684.63 (773.19)	1780.23 (892.76)	1702.91 (759.70)	0.192
ATP differences (before-after)	1133.76 (751.99)	1189.27 (703.27)	0.610	988.48 (622.60)	1279.04 (844.41)	1200.07 (690.03)	1178.47 (723.89)	0.276
ATP improvement rate (better+)	0.09 (1.59)	0.26 (0.51)	0.315	0.30 (0.24)	-0.13 (2.23)	0.35 (0.30)	0.18 (0.65)	0.223

Notes: Hand cleanliness is measured using an ATP luminometer device which detects actively growing microorganisms by measuring the light emitted from the reaction of ATP with the firefly enzyme luciferase. Although smaller figures suggest cleaner hands, i.e., less microorganisms, there are large individual variations in the figures. Thus, tests were conducted before and after handwashing to measure whether appropriate handwashing had been done. The ATP improvement rate was calculated as (ATP-before – ATP-after)/ATP-before, making the larger the figure the higher the improvement. HE-school values exhibit cleaner hands and higher improvement rate, however, statistical differences are not detectable due to large standard errors, possibly due the small sample size. Child-level data which were collected from 10% of pupils are averaged per school. The actual numbers of child-level observations are for HE-intervention: non-HE school (N=430); HE-school (N=437), and for Cross-cutting HESP-intervention: control school (N=216); SP-school (N=214); HE-school (N=216); HESP-school (N=221). See Omura (2024) [30] for the child-level analysis using ATP measure.
